# Early Administration of Therapeutic Anticoagulation Following Intravenous Thrombolysis for Acute Cardiogenic Embolic Stroke Caused by Left Ventricular Thrombus: Case Report and Topic Review

**DOI:** 10.3389/fneur.2015.00009

**Published:** 2015-02-02

**Authors:** Rick Gill, Elisabeth Donahey, Sean Ruland

**Affiliations:** ^1^Department of Neurology, Loyola University Medical Center, Maywood, IL, USA; ^2^Department of Pharmacy, Loyola University Medical Center, Maywood, IL, USA

**Keywords:** acute ischemic stroke, cardioembolic, thrombolysis, left ventricular thrombus, anticoagulation, rtPA, alteplase, pharmacokinetics

## Abstract

Cardiogenic cerebral embolism represents 20% of all acute ischemic strokes (AISs) with one-third of these being caused by left ventricular thrombus (LVT). LVT is not a contraindication for treatment with intravenous recombinant tissue plasminogen activator (IV rtPA) for AIS. However, the subsequent treatment of a potentially unstable LVT is contraindicated for 24 h following the use of IV rtPA according to current guidelines. We present a 66-year-old man with AIS treated with IV rtPA. Echocardiogram shortly after treatment demonstrated both a large apical and septal thrombus in the left ventricle and at 12 h post IV rtPA infusion, therapeutic anticoagulation with heparin was started without complication. In practice, the action of IV rtPA outlasts its apparent half-life because of thrombin-binding and the prolonged effects and longer half-life of its product, plasmin; however, the pharmacokinetics do not warrant prolonged avoidance of therapeutic anticoagulation when clinically indicated. Our case demonstrates that anticoagulation for potentially unstable LVT can be safely initiated at 12 h following IV rtPA treatment for AIS.

## Introduction

A 66-year-old man presented to our hospital with acute left sided weakness, facial droop, slurred speech, and complete left hemianopsia (NIHSS 6) within 90 min of symptom onset. His medical history was remarkable for ischemic stroke a year prior with no residual deficit and myocardial infarction (MI) 15 years prior complicated by ventricular fibrillation requiring temporary balloon pump. He had been discharged earlier that day from another hospital where evaluation for shortness of breath revealed a left ventricular thrombus (LVT). He was to initiate warfarin that evening, which he had not yet commenced. The patient had no contraindications to intravenous recombinant tissue plasminogen activator (IV rtPA) and was treated within 60 min of presentation.

**Figure 1:**
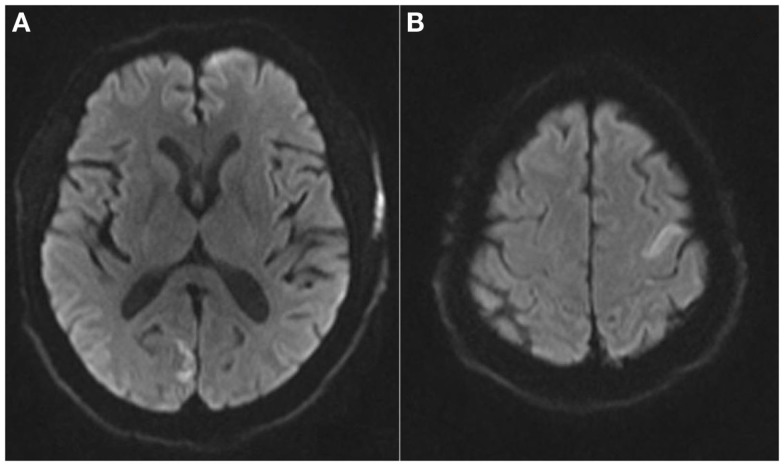
**Magnetic resonance imaging brain axial diffusion weighted images demonstrating areas of restricted diffusion in the R occipital lobe (A) and left superior frontal lobe (B)**.

**Figure 2:**
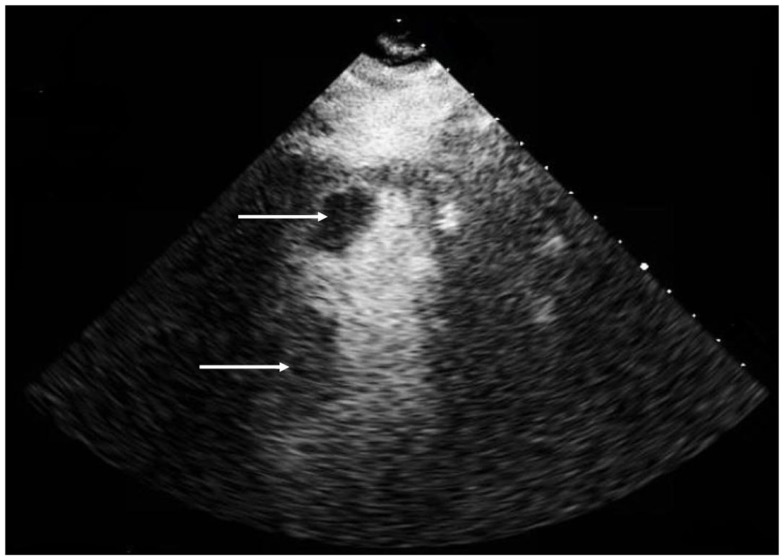
**Transthoracic echocardiogram, apical 2 chamber view with contrast demonstrating a left ventricular apical thrombus (2.4 cm × 1.4 cm) (top arrow) and a second thrombus (1.6 cm × 2.2 cm) (bottom arrow) adherent to the interventricular septum**.

Magnetic resonance imaging (MRI) demonstrated acute infarcts in both cerebral hemispheres (Figure 1) and no evidence of hemorrhage. An apical LVT (2.4 cm × 1.4 cm) and a second thrombus adjacent to the interventricular septum (1.6 cm × 2.2 cm) were confirmed on transthoracic echocardiogram (TTE) (Figure 2) within 12 h of presentation and 10 h of completion of IV rtPA infusion. Ejection fraction was 25%; regional variation of wall motion and akinesis of the left ventricle (LV) were also noted.

This presented a therapeutic conundrum whereby the presence of a potentially high recurrent cardioembolic source required anticoagulation but was contraindicated due to recent administration of IV rtPA. The patient’s activated partial thromboplastin time (aPTT), prothrombin time (PT), and international normalized ratio (INR) were verified to be within the normal range. The pharmacokinetics of IV rtPA and its byproducts were reviewed and the decision to start therapeutic anticoagulation was made. Non-bolus IV heparin infusion was initiated 12 h after IV rtPA infusion. The patient’s neurological deficit improved and he was bridged to warfarin and discharged home with a favorable outcome (NIHSS 1, modified Rankin Scale 1).

## Discussion

### LVT and cardiogenic embolism

Cardiogenic embolism causes 20% of all ischemic strokes. While non-valvular atrial fibrillation (AF) is responsible for approximately 50% of these cases, left ventricular mural thrombus represents almost one third of cases (1). The majority of LVT have been associated with acute myocardial infarction (AMI), and also occur in patients with chronic ventricular dysfunction from coronary artery disease, hypertension, or dilated cardiomyopathy (1). Myocardial injury can lead to severe regional wall motion abnormality and hypokinesis, akinesis, dyskinesis, or aneurysmal dilatation of the LV. Patients with large anterior MI have a high risk of developing LVT and subsequent cardiogenic thromboembolism (2). The incidence of embolic complications is highest in the first 1–3 months post-MI, with an absolute risk of 9% in patients with acute MI complicated by LVT (3). Beyond the acute phase, patients with persistent myocardial dysfunction have a 5-year stroke risk of 8% (4, 5). TTE is 23% sensitive and 96% specific for LVT detection (6), improving to 92% sensitivity when a high prevalence of LVT was anticipated (7) and remains the preferred method for detection. If image quality is poor or LVT risk is high with negative or equivocal echocardiography, contrast enhanced cardiac MRI (CMR) should be considered. It has a sensitivity of 88% and specificity of 99% (6).

The 2013 American College of Cardiology Foundation/American Heart Association Guideline for the Management of ST-elevation MI recommend vitamin K antagonist therapy for 3 months (2, 8) and perhaps indefinitely (9) with a goal INR of 2.0–3.0 if the risk of thromboembolism exceeds the bleeding risk when LVT is demonstrated. This recommendation was based on observational studies reporting better outcomes and fewer cerebral emboli in patients treated with heparin and then transitioned to warfarin. No guideline for prolonged duration of anticoagulation exists and the decision is guided by follow-up echocardiography and demonstration of resolution or persistence of the clot and embolic risk as determined by the clinician.

Cardiomyopathy leading to impaired LV systolic function and reduced stroke volume creates relative stasis and risk for LVT development (5). Etiology of cardiomyopathy can be ischemia or infarction, or non-ischemic causes such as genetic or acquired structural or metabolic defects. Decreased ejection fraction and older age are both independent risk factors for stroke after MI (4). The warfarin versus aspirin in reduced cardiac ejection fraction (WARCEF) trial showed fewer ischemic strokes in follow-up for patients in sinus rhythm and ejection fraction <35% randomized to warfarin compared to aspirin 325 mg daily; however, the primary end point of ischemic stroke, intracerebral hemorrhage, or death was similar between groups (10). If LV dysfunction due to extensive regional wall motion abnormalities is demonstrated, prophylactic treatment with warfarin may be considered in patient with previous stroke or transient ischemic attack (TIA) (1). Similarly, in patients with LVT in the setting of congestive heart failure due to dilated cardiomyopathy, anticoagulation might be reasonable particularly if the embolic potential is high and the bleeding risk is low. This is supported by indirect data from observational studies in patients with LVT after MI (11).

No single clinical feature predicts embolic risk. Thrombi that demonstrate mobility, protrusion, and central echolucency are higher risk (12). A theoretical risk of embolization from destabilization of a LVT after thrombolysis exists. However, only case reports exist of post thrombolysis infarction in the contralateral hemisphere (13). The largest case series of five patients did not demonstrate subsequent re-embolization after IV thrombolysis (14). The presence of LVT is not listed as a contraindication to IV thrombolysis for acute ischemic stroke (AIS) according to current guidelines (15).

### IV rtPA kinetics

Alteplase was FDA-approved for use in acute stroke in 1996 and is also indicated for use in pulmonary embolism (PE) and AMI (16, 17). It exerts its thrombolytic effects by converting plasminogen to plasmin on clot-bound fibrin, in turn degrading fibrin clot (18). This results in a 16–36% decrease in circulating fibrin and 16–62% decrease in fibrinogen (19). Alteplase has limited activity in the absence of fibrin (20).

The elimination of alteplase is biphasic, reflecting a two-compartment circulatory model (21–23). In healthy patients, there is an alpha-phase with rapid decrease in plasma concentration due to tissue distribution. The alpha-phase half-life (*t*1/2) ranges from 3.3 to 6 min. The beta-phase is a slower decrease in plasma concentration due to metabolism and excretion with a *t*1/2 of 26–40 min (24, 25). In patients with AMI, the alpha-phase *t*1/2 of alteplase is <5 min and the beta-phase *t*1/2 is 16–88 min (23, 26, 27). The majority of alteplase pharmacokinetic studies have been conducted in AMI patients, who may have different elimination characteristics compared to patients with AIS (18). The mean age in many AMI studies is <60 years, and >45% of patients who receive alteplase for AIS are >70 years (18, 28). Alteplase metabolism may be slower in the elderly, contributing to increased hemorrhage risk (18, 29). Additionally, alteplase doses used in AMI studies differ from the 0.9 mg/kg dose for AIS.

After alteplase administration, fibrinogen concentrations return to 81% of pre-infusion values within 24 h (19, 23, 30). Plasminogen and alpha-2 antiplasmin demonstrate similar concentration trends (70 and 35% of pre-infusion concentrations at 2 h post-infusion, 83 and 88% at 24 h, respectively) (23). Fibrin degradation products are found for upwards of 7 h in patients treated with alteplase (31). Therefore, safe timing of antithrombotic initiation after alteplase treatment is uncertain. A study of 60 AIS patients who received IV rtPA followed by 2850 units nadroparin twice daily either immediately after rtPA or 24 h later found similar symptomatic intracranial hemorrhage rates at 36 h between the two groups (early group 8.6 and 4% later group OR 1.8, 95% CI 0.5–3.2) (32). Sixteen (45.7%) early treatment and nine (36%) standard treatment patients achieved a modified Rankin Scale score of 0 or 1 at 3 months (OR 1.2, 95% CI 0.5–3.2) (32). Patients with PE are generally treated with thrombolysis followed by immediate anticoagulation with heparin, and in a trial comparing this regimen to heparin alone, only one intracranial hemorrhage was observed, in a patient who had sustained head trauma (33). Larger trials investigating early versus delayed anticoagulation for AIS patients who have received IV rtPA are needed.

## Conclusion

Left ventricular thrombus can complicate both AMI and CHF with dilated cardiomyopathy. These patients often have overlapping risk factors for stroke and additional factors must be considered in the management of AIS in this population. Additionally, with increasing efficiency and expedited care at advanced stroke centers, ventricular thrombi may be increasingly discovered very early in the patient’s post stroke care. LVT is not a contraindication for treatment of AIS with IV rtPA. However, subsequent use of therapeutic anticoagulation following thrombolysis within 24 h is contraindicated. This case demonstrates that early anticoagulation, 12 h post-thrombolytic infusion, can be safely administered. Further evaluation of the safety of early anticoagulation for LVT with high recurrent embolic potential following IV rtPA for AIS is needed.

## Conflict of Interest Statement

The authors declare that the research was conducted in the absence of any commercial or financial relationships that could be construed as a potential conflict of interest.
